# Supplementary material for: Health and wellness in the Australian coal mining industry: A cross sectional analysis of baseline findings from the RESHAPE workplace wellness program
Source: PLoS One. 2021 Jun 4;16(6):e0252802. doi: 10.1371/journal.pone.0252802 (PMC8177661; doi:10.1371/journal.pone.0252802)
Supplement: Attachment — Submitted filename: Response to reviewer_R1_Final.docx [file pone.0252802.s001.docx]

Response to reviewer’s table:

Dear Associate Professor Spradley,

Thank you for the opportunity to revise manuscript PONE-D-21-05421 entitled: *“Health and wellness in the Australian coal mining industry: A cross sectional analysis of baseline findings from the RESHAPE workplace wellness program”.*

Also, we thank the reviewers for their constructive critique of the manuscript. We believe the suggested changes have greatly improved the quality of the manuscript. Please see below a table addressing each of the reviewer comments, changes to the manuscript and location.

| **Reviewer comment** | **Response to reviewer** | **Changes to manuscript** | **Location of changes** |
| --- | --- | --- | --- |
| I know space is limited, but a bit more information about the RESHAPE program would help the abstract. | Comment received with thanks. We have added an additional sentence to the abstract to explain a little more of the RESHAPE framework. | Added: “RESHAPE is an eight-step framework (based on the WHO ‘Health Workplace Framework and Model’) which aims to provide a sustained approach to wellness in the workplace.” | Abstract, page 2, line 34-35 |
| The phrase “5 serves of vegetables” in the abstract needs to be corrected. | Thank you for your feedback. We have amended this phrase within the manuscript for readability purposes. | Removed: ~~“the recommendation of a minimum of five serves of vegetables”~~  Added: the daily recommendation for vegetables (5 serves) | Abstract, page 2, line 43 |
| What is novel about this study. Any similar studies in a comparative cohort from other countries? And what about this specific industry linked to greater rates of overweight/obesity versus other labor industries? | Novel aspects of this study include the RESHAPE framework. RESHAPE does not prescribe a certain wellness intervention or one size fits all mantra, rather allows the organisation to choose the wellness initiative based on their needs and policies.  Another similar study comparative to some of the objectives of RESHAPE include the POWERPLAY study by Johnson et al, (2016) which used a workplace diet and physical activity intervention to achieve weight loss in men in Canada.  Regarding the factors which make mining more susceptible to weight gain, these have been developed and explored further in the background of the manuscript. | Added: “The mining industry faces many unique occupational factors which promote higher body mass index (BMI) values compared to other labour industries. The sector is heavily male dominated with higher than average rates of obesity found in men; (4) (23) mining jobs often utilise shift work which has been identified as a risk factor for excessive weight.(24) and mining jobs are often found in regional and remote areas which experience greater obesity rates compared to metropolitan areas;(23) In addition, the location of workplaces are often located in rural and/or remote areas that may require a challenging commute to work and a degree of physical and social isolation.(25)” | Background, page 6. Line 133-139 |
| The introduction is too long and should focus on the rationale for this study – it should be 2 pages at most. Instead providing “baseline data” as a rationale for conducting this study, a directional hypothesis statement needs to indicate what is expected about diet and physical activity programs on rates of overweight and obesity in blue-collar workers. | Thank you for your comments. We have streamlined the introduction to keep information in line with the rationale of the study. We have also added a hypothesis as requested. The Authors would like to highlight that the section: “What is RESHAPE”, has been streamlined and moved to the methods section. We believe this section is vitally important in understanding the aims of the study and how RESHAPE is different from other workplace wellness programs. | Removed:  ~~Overweight and obesity as defined by the World Health Organisation (WHO) is abnormal or excessive fat accumulation that increases the risk of certain diseases.(1) Body Mass Index (BMI) is one way to assess whether a person’s body weight is within a healthy range. BMI is a person’s weight in kilograms divided by the square of height in meters, with higher values typically being an indicator of greater body fatness.(5) For classification purposes, BMI is divided in ranges to represent a person’s body fatness and are as follows; BMI <18.5 underweight range; 18.5 to <25 healthy weight range; 25 to <30 overweight and 30.0 or higher obese.(5)~~  ~~There are, however many barriers to workplace health promotion (WHP) that need to be overcome for successful implementation.(8) Time and knowledge on how to effectively implement WHP are two common barriers shared by both small and large scale organisations.(8) One potential solution to overcoming the knowledge obstacle are gender targeted interventions which can play an underlying role in the receptiveness and sustainability of a WHP program.(11, 12) Johnson et al.,(13) in their gender targeted POWERPLAY study showed encouraging acceptability of male participants, achieving a retention rate of 92% between the two time points (baseline to 6 months). And whilst fruit and vegetable consumption did not increase over the study duration, moderate to vigorous physical activity did by 112.3 min/week.(13) This sentiment of men favouring physical activity over nutrition interventions is one that is echoed in the literature (14) and underscores the benefits of gender targeting health promotion initiatives.~~  ~~Restraints related to the amount of time employees are at work also negatively implicates the implementation of WHP.(15) This can be overcome with organisational commitment for staff to actively be involved in programs or enabled to make healthier decisions during the work day.(16) This can be challenging for workers who are employed in shift work as shift rostering may mean programs are not run when the employee is at work. Although gender targeted WHP is in its infancy, results have been positive, particularly in achieving weight loss in male dominated industries.(17) There is potential for blue-collar male dominated industries, such as mining to benefit from gender targeted, workplace specific, weight loss interventions as this population is often underrepresented in health research, yet may benefit the most from such programs.(18)~~  ~~The fly-in, fly-out (FIFO) or drive-in, drive-out (DIDO) nature of the job can also have negative consequences on the physical and mental health of workers.(26) Those who work in the mining industry have also been shown to have higher than population average levels of psychological distress (p <0.001) compared to an age and gender matched sample.(27) In addition, for males in the mining industry, the risk of hazardous or harmful drinking is nearly twice as likely than gender-matched community levels (24% compared to 45.7% respectively).(28)~~  ~~The mining industry faces a unique set of challenges to the implementation of successful workplace wellness and health promotion initiatives. This sector is typically male-dominated,(19) involves shift work, long shifts (12 hours) and rotating rosters (disrupting sleep and circadian rhythms).~~  ~~To reshape something means to change its structure or organisation. The word RESHAPE not only aligns with the vision ‘to lose 1kg at a time, one miner at a time, one year at a time’, it also reflects the changes required to achieve this goal at an organisational and individual level~~.  Rewritten:  The Australian mining sector maintains a strong emphasis on safety however there has been less emphasis on health as part of workplace health and safety until recent years. One of these recent developments was the: ‘Blueprint for the management of overweight and obesity in the NSW mining industry’ which was developed in response to an industry identified need. (14) The blueprint highlighted the need for RESHAPE, which was conceived by a sub-committee of the New South Wales Minerals Council (NSWMC) Work Health Safety Committee. This committee developed the framework for a coordinated plan of action for mining operations to engage in industry-wide change to prevent and manage obesity. RESHAPE is an eight-step framework which aims to provide a sustained approach to wellness in the workplace.  The mining industry faces many unique occupational factors which promote higher BMI values compared to other labour industries. The sector is heavily male dominated with higher-than-average rates of obesity found in men; (4) mining jobs often utilise shift work which has been identified as a risk factor for excessive weight;(15) as well as mining jobs are often found in regional and remote areas which experience greater obesity rates compared to metropolitan areas.  Considering the aforementioned factors, this study aims to implement the RESHAPE framework into three Australian coal mining sites. It is hypothesised that the RESHAPE framework will lead to greater wellness outcomes for the individual (weight loss) compared to once off wellness initiatives which are current practice in this industry. This paper presents baseline findings from a pilot study that implements RESHAPE and investigates the issue of overweight and obesity in the coal mining industry. Knowledge gaps remain alongside concerns the success of diet and physical activity programs within blue-collar workers, particularly miners. This paper looks to bolster this shortcoming of the literature base and provides discourse concerning wellness in blue-collar male dominated industries. | Edits throughout background and start of methods, pages 4-10. |
| As mentioned in the introduction, is there any specific information about the workplace health promotion programs? How does this study help overcome time and knowledge and gender targeted interventions to implement workplace health programs? | Thank you for your comments. As this is the baseline paper, specifics regarding the type of wellness program chosen by the individual sites have not been included in this paper and will feature in future papers when discussing follow-up data. With regards to topics of gender targeting and overcoming barriers for implementing wellness programs, these have been omitted from the background to reduce the word count. These themes will be for future discussion. |  |  |
| In the conclusions, it is stated, “Poor nutrition is likely to be a key component in this scenario; however, this is further compounded by workplace factors which exacerbate challenges workers face in implementing health behavior change.” What are these factors, and can they be assessed here? | Comment received with thanks. Workplace factors such as shift work status can implicate dietary habits. In the discussion we explored these themes and assessed shift status as part of the baseline survey. We have added this information for greater clarity. | Added: “(e.g., participation in shift work)” | Page 27, conclusions  Line 545 - 546 |
| RESHAPE is not mentioned much in the discussion. | We have added some further discourse to the discussion about the RESHAPE framework. | The following has been added to the discussion:  The goal of RESHAPE however is not to implement a one off wellness program. Rather, bring about organizational change and the cultivation of an environment which values the health of its employees. This long-term success does not simply depend on the contents of the program; instead the models used to action the intervention can implicate health outcomes at an individual level.(72) The RESHAPE framework takes a non-traditional approach to workplace wellness and looks at organizational change as opposed to a solely focusing on the individual.(73) This is evident by step three of the RESHAPE framework which looks to align the RESHAPE charter to organizational principles. This step is important for an organization as it mandates that health and wellness will be a part of the ethos of the establishment going forward, and clearly articulates to employees that their health and wellness is a priority. Without this organization change it is unlikely that the workplaces will cultivate any meaningful change with regards to the health and wellness of their employees.(74)  Despite the theoretical relevance of sustainably enacted wellness programs in the workplace, there exists a dearth of literature concerning this subject matter.(74) This may be attributed the degree of difficulty of continuously implementing programs that are effective. Jenny and colleagues (75) in their study concerning process and outcome evaluations of a workplace stress management program further ratify this notion; that the successful implementation of workplace programs requires several overarching themes including: perseverance, strong coalitions, constant fine-tuning and support, systematic training and reflection. Once again circling back to the necessity of a framework over the contents of the program, RESHAPE incorporates all these aspects within its eight steps; and looks to apply these theories within the workplace for the good of the individual and organization as a collective whole. | Page 27, discussion. Line 562 - 584 |
| Why not include figures about physical activity? | We have completed some additional analysis around the relationship between physical activity and obesity including regression models, as well as including a figure comparing physical activity minutes between mining populations and the general Australian population. Furthermore, we explored themes of physical activity in relation to workplace wellness in the discussion section of the manuscript. |   The following has been added:  Interestingly total physical activity minutes in the last week showed a significant relationship between 150-to-300-minute category and increased BMI (B=1.81, 95% CI: 0.139, 3.485, p= .034).  INSERT FIGURE 3 ROUGHLY HERE  Figure 3 depicts a comparison of total physical activity in the last week between participants and the general Australian population, taken from the 2017-18 National Health Survey.(4) Participants in the study faired similar to the general Australian population with roughly 60% completing 150 minutes of physical activity or more over the week.  In the discussion the following has been added:  Physical activity plays an important role in weight homeostasis. (55) Interestingly within our sample, participants who belonged to the 150 to 300 minutes category of physical activity showed a significant relationship with increasing BMI. Whilst recommended levels of physical and increasing BMI is rather contradictory, this could be explained by sex preferences towards certain methods of weight loss. Within masculinity research, it has been shown that men have a greater affinity towards physical activity as opposed to dieting to illicit weight loss.(43) As the sample is 90% males, this may offer an explanation for this phenomenon; that men are increasing physical activity in response to excess weight, albeit, without success, as the consequential effects of a bad diet can at times out weight increased exercise.(56) This is further reinforced within the literature, with studies reporting that physical activity alone has a diminished effect of weight balance (when compared to diet alone and a combination of diet and physical activity).(57, 58) Furthermore, the intensity of exercise rather than minutes active has been shown to be more influential with regards to weight maintenance.(57) Exploration of this using objective measures (e.g. accelerometer or heart rate trackers) would help affirm this notion.  Workplace health promotion programs, particularly amongst men, who utilized both diet and exercise components have shown success.(59) One example of a study which incorporated both these themes was the Preventing Obesity without Eating like a Rabbit (POWER) by Morgan et al., (60) In a two-armed randomized control trail of 110 overweight or obese male employees at a metal manufacturing plant, significant changes (P <0.001) to weight (-4.3 kg), waist circumference (-5.9cm) and BMI (-1.4), were observed all favoring the intervention group. Using a multi-component intervention that focuses on both diet and physical activity may produce a greater effect (with regards to weight outcomes) for employees participating in a workplace health promotion program.  Removed  ~~The inadequate consumption of fruit and vegetables in both this mining population and the general Australian population is a significant issue for health. The preventive effect of fruits and vegetables regarding noncommunicable disease such as obesity, cardiovascular disease, type 2 diabetes and certain cancers is endorsed by the WHO, and empirically supported within population research and modelling.(57, 58) One such study by Yu et al.,(57) cross-sectionally assessed 26,340 individuals in Canada, and found that one standard deviation increase of total fruit and vegetable intake was inversely associated with waist circumference, percentage fat mass, fat mass and BMI ((−0.12 kg/m2; 95% CI −0.19 to –0.05), (−0.40 cm; 95% CI −0.58 to –0.23), (−0.30%; 95% CI −0.44 to –0.17) (−0.14 kg/m2; 95% CI −0.19 to –0.08) respectively). This illustrates that often small changes to diet can have substantial positive impacts to health.~~ | Table 1, page 12  Table 4, page 17  Page 18, Line 327 – 329  Page 19, line 351 - 355  Discussion, page 23-24, Line 444-479  Discussion, page 25-26, Line 513-522 |
| Including a biostatistician would be helpful and provide rigor. | Comment received with thanks. The team have sufficient expertise in the statistical methods conducted in this manuscript, which has been supported by statisticians. If required, a letter from statistician can be provided upon request from the editor. |  |  |
| As funding was provided from a coal industry, is this some type of conflict of interest? | Thank you for you queries. The funding from the Coal Industry has been declared in a transparent nature. The research has been undertaken by University researchers independent of the Coal Industry and is undergoing a process of independent peer review through the process of publication. |  |  |
